# Three-dimensional hydrogel with human Wharton jelly-derived mesenchymal stem cells towards nucleus pulposus niche

**DOI:** 10.3389/fbioe.2023.1296531

**Published:** 2023-12-12

**Authors:** Isma Liza Mohd Isa, Izzat Zulkiflee, Raed H. Ogaili, Nurul Huda Mohd Yusoff, Natasya Nadia Sahruddin, Shaiful Ridzwan Sapri, Elvy Suhana Mohd Ramli, Mh Busra Fauzi, Sabarul Afian Mokhtar

**Affiliations:** ^1^ Department of Anatomy, Faculty of Medicine, Universiti Kebangsaan Malaysia, Kuala Lumpur, Malaysia; ^2^ CÚRAM, SFI Research Centre for Medical Devices, University of Galway, Galway, Ireland; ^3^ School of Medicine, University of Galway, Galway, Ireland; ^4^ Centre for Tissue Engineering and Regenerative Medicine, Faculty of Medicine, Universiti Kebangsaan Malaysia, Kuala Lumpur, Malaysia; ^5^ Department of Orthopaedics and Traumatology, Faculty of Medicine, Universiti Kebangsaan Malaysia, Kuala Lumpur, Malaysia

**Keywords:** intervertebral disc (IVD) degeneration, low back pain, hydrogel, nucleus pulposus, Wharton jelly mesenchymal stem cells

## Abstract

**Introduction:** A regenerative strategy employing extracellular matrix (ECM)-based biomaterials and stem cells provide a better approach to mimicking the three-dimensional (3D) microenvironment of intervertebral disc for endogenous tissue regeneration. However, there is currently limited understanding regarding the human Wharton Jelly derived-mesenchymal stem cells (hWJ-MSCs) towards nucleus pulposus (NP)-like cells. Our study focused on the development of 3D bioengineered hydrogel based on the predominant ECM of native NP, including type II collagen (COLII) and hyaluronic acid (HA), which aims to tailor the needs of the microenvironment in NP.

**Methods:** We have fabricated a 3D hydrogel using from COLII enriched with HA by varying the biomacromolecule concentration and characterised it for degradation, stability and swelling properties. The WJ-MSC was then encapsulated in the hydrogel system to guide the cell differentiation into NP-like cells.

**Results:** We successfully fabricated COLII hydrogel (2 mg/ml) and HA 10 mg/ml at a weight ratio of HA and COLII at 1:9 and 4.5:9, and both hydrogels physically maintained their 3D sphere-shaped structure after complete gelation. The higher composition of HA in the hydrogel system indicated a higher water intake capacity in the hydrogel with a higher amount of HA. All hydrogels showed over 60% hydrolytic stability over a month. The hydrogel showed an increase in degradation on day 14. The hWJ-MSCs encapsulated in hydrogel showed a round morphology shape that was homogenously distributed within the hydrogel of both groups. The viability study indicated a higher cell growth of hWJ-MSCs encapsulated in all hydrogel groups until day 14.

**Discussion:** Overall, our findings demonstrate that HA/COLII hydrogel provides an optimal swelling capacity, stability, degradability, and non-cytotoxic, thus mimics the NP microenvironment in guiding hWJ-MSCs towards NP phenotype, which is potentially used as an advanced cell delivery system for intervertebral disc regeneration.

## Introduction

Low back pain (LBP) is a major unmet clinical need that imposes the highest amount of disability worldwide (Wu et al., 2020). The cause of LBP is multifactorial, but disc degeneration is considered the primary aetiological factor, accounting for between 26% and 42% of patients with LBP (Cheung et al., 2009). The intervertebral disc (IVD) is a fibrocartilage connecting vertebral bones of the spine that mainly provides mechanical support and allows movement flexibility. The IVD consists of a gelatinous proteoglycan-rich nucleus pulposus (NP) in the central region that is surrounded by collagen-rich lamellae of annulus fibrosus (AF) and superiorly confined with the cartilage end plate. In humans, the healthy NP contains large and vacuolated notochordal cells (NCs) and maintains a high proteoglycan content. Nevertheless, the appearance of NCs is gradually replaced by small chondrocyte-like cells as early as adolescence (Rodrigues-Pinto et al., 2018). Therefore, the phenotypic change in the NP is associated with the onset of disc degeneration, which starts in young adult, progresses with ageing and is further advanced by pathological insults (Joyce et al., 2021).

Degeneration of the intervertebral disc is characterised by an imbalance of extracellular matrix (ECM) homeostasis that contributes to loss of water content, a decrease of cellularity, an increase of inflammation, disc collapse and AF tear, resulting in mechanical instability. The unique avascularity and metabolic restriction result in the disc being poorly innervated; however, an increase of pro-inflammatory cytokines and neurogenic mediators eventually promotes vascularisation and nerve ingrowth into the degenerated disc, thereby inducing sensitization of nociceptive nerve fibres and results in discogenic pain (Lyu et al., 2021). Critically, current treatments include conservative management using pharmacological agents with a last resort of surgical intervention that aims to alleviate pain; however, none of these treatments could restore the disc anatomically and promote tissue regeneration (Prithvi Raj, 2008). Surgery is the final option when conservative treatments fail after few months, and it is also necessary if degenerative disc disease leads to issues like pain while walking, muscle weakness, unusual sensations in the lower body, and progressing nerve problems that affect daily activities (Mohd Isa et al., 2023). Therefore, a tissue engineering strategy is required as an alternative approach not only to alleviate pain but also to facilitate disc regeneration (Mohd Isa et al., 2022).

Tissue engineering employing biomaterials is promising as it allows for a wide selection of biomaterials to tailor the needs of the microenvironment, while in the advanced stage, it could be combined with exogenous precursor cells such as progenitor, notochordal and stem cells to instruct cellular function towards the desired phenotype and re-populate disc cellularity for intrinsic tissue regeneration (Mohd Isa et al., 2018a). Specifically, biocompatible ECM-based biomacromolecules give more advantages as they are non-cytotoxic in nature and can provide instructive cues through the regulation of cellular signalling for tissue development, maintenance, and regeneration. Therefore, using biomaterials that are a biological response to cells is useful to mimic the healthy NP microenvironment to promote tissue regeneration. Notably, type II collagen (COLII) is among the biomacromolecules that are highly abundant in the NP and has ligands with the cell’s transmembrane receptor, such as integrin, to regulate intracellular signalling, and eventually could facilitate a down-stream effect in modulating cytoskeletal organisation and differentiation of precursor cells towards the NP phenotype, which is desirable for tissue regeneration. Type II collagen has also been used to model three-dimensional (3D) *in vitro* disc inflammation (Srivastava et al., 2017). Besides, glycosaminoglycan (GAG) is also a major biomacromolecule in the NP that plays a pivotal role in maintaining ECM homeostasis and disc hydration. For example, hyaluronic acid (HA) is known to exhibit anti-inflammatory (Mohd Isa et al., 2015; Inoue et al., 2021; Kotla et al., 2023), anti-nociceptive and anti-catabolic effects that are therapeutically necessary for promoting disc repair *in vivo* (Mohd Isa et al., 2018b).

In the design of biomaterials, the scaffold is usually chemically crosslinked and/or in combination with other types of biomaterials to obtain a highly stable scaffold that has an appropriate viscoelastic property to resist biomechanical loads. The hydrogel-based 3D scaffold is widely studied in disc tissue engineering due to the hypoxic microenvironment in the NP. Because of the lower cellularity of the disc, an exogenous cell encapsulated scaffold is employed to promote intrinsic repair by increasing cell population through cell differentiation and paracrine secretion to maintain the cellular niche for long-term tissue regeneration. Incorporating pluripotent or mesenchymal stem cells with biomaterials is attractive due to their high accessibility, multilineage differentiation capability and low immunogenicity. In the context of bone marrow derived-MSC treatment, disc height was restored to a remarkable 95%–100%, as well as flexion/extension and lateral bending stiffness, the restoration of GAG levels, decreased expression of catabolic genes (MMP2/3/9/13, ADAMTS4/5) and improved histopathological assessment in IVDs of the ovine model (Shu et al., 2018). The safety and efficacy of mesenchymal precursor cells (MPCs) in HA to treat degenerative disc patients with low back pain have been completed in phase II clinical trials (Study Details, 2020). Nevertheless, little is currently known about the human Wharton Jelly-derived mesenchymal stem cells (hWJ-MSCs) phenotype towards NP-like cells. The strategy to incorporate hWJ-MSCs with biomaterials is attractive due to their high accessibility, multilineage differentiation capability and low immunogenicity. WJ-MSCs can be obtained from umbilical cords, highly accessible in large quantities from millions of births globally without causing donor morbidity, which is one of the best sources of allogeneic MSCs for MSC therapy (Liau et al., 2020). The MSCs produced from Wharton’s jelly are not as diverse as adult MSCs and are hypoimmunogenic and nontumorigenic, but they are easily separated and grown, have a high proliferation rate and can maintain their stemness qualities for multiple passages *in vitro* (Bongso and Fong, 2013). WJ-MSCs may reduce the risk of immunological rejection because they often display low levels of major histocompatibility complex (MHC) class I and lack MHC class II expression (Liu et al., 2012).

Herein, this study aimed to develop a novel bioengineered 3D construct of HA/COLII- hydrogel to mimic the NP niche in supporting the delivery of pre-conditioning hWJ- MSCs to promote disc regeneration. It is hypothesised that a hydrogel-based 3D construct fabricated from HA and COLII mimicked the NP niche in guiding MSC differentiation towards NP-like cells by varying the biomacromolecule concentration *in vitro*. The implication of these findings may imply the use of 3D hydrogel as IVD-specific matrices by mimicking native ECM of IVD to maintain cellular phenotype, as an advanced cell delivery system to support stem cell transplantation for IVD regeneration, as well as potential therapeutic biomaterials targeting mechanisms underlying IVD degeneration in the treatment of low back pain, which in line with biomaterials for IVD tissue engineering towards a precision medicine approach.

## Results

**FIGURE 1:**
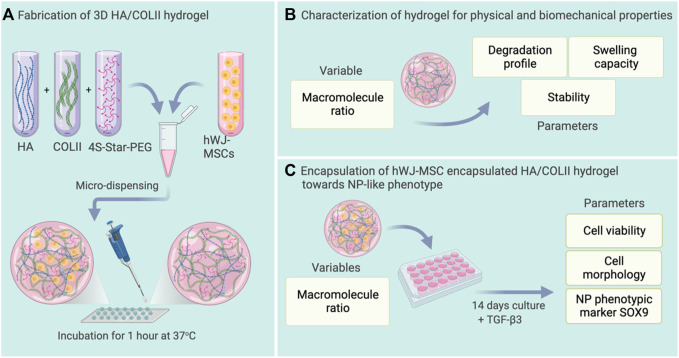
Schematic representation of the experimental plan. **(A)** Fabrication of HA/COLII hydrogel with or without microencapsulation of hWJ-MSCs. **(B)** Characterization of hydrogel for physical and biomechanical properties. **(C)** Differentiation of hWJ-MSC encapsulated in HA/COLII hydrogel towards NP-like phenotype *in vitro*. Created with BioRender.com.

Figure 1 illustrates the fabrication of the hydrogels, and experimental plans of hydrogel characterization for swelling capacity, stability, and degradation properties. The hydrogels were then utilized for cell encapsulation with hWJ-MSCs for differentiation towards NP-like cells.

**FIGURE 2:**
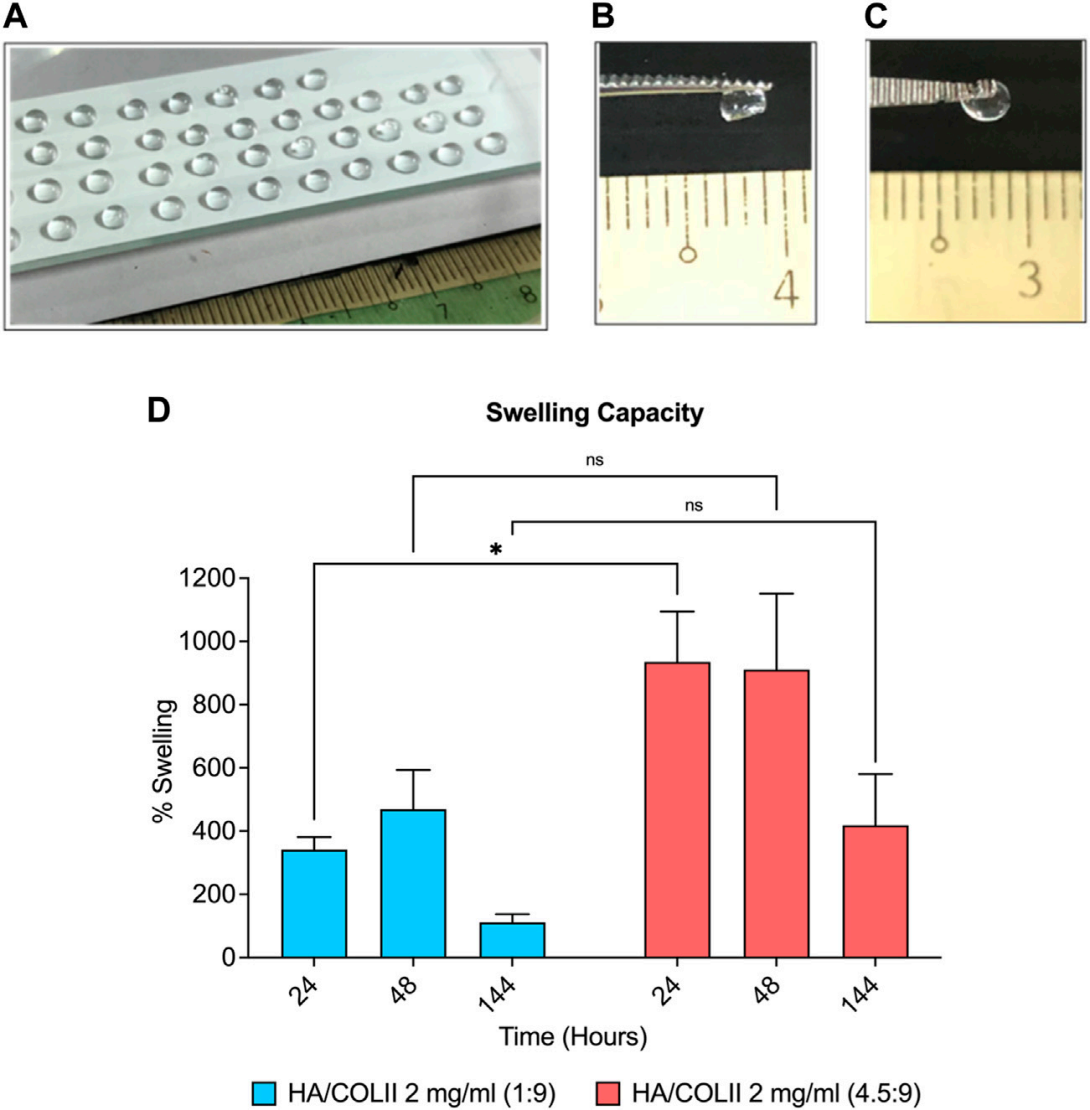
Formulation and characterisation of HA/COLII hydrogel. **(A)** Photograph of micro-dispensing of spherical-shaped hydrogels on the modified hydrophobic surface of glass slide at room temperature. Hydrogels maintained a 3D spherical shape after complete crosslinking at 37°C in both formulations at 1:9 **(B)** and 4.5:9 **(C)** weight ratios of HA and COLII. **(D)** Swelling of the hydrogels in PBS. *p < 0.05, the significant difference between groups, by two-way ANOVA and Bonferroni’s *post hoc* test. Data are means ± SEM, *n* = 3. Scale bar in mm.

The 3D hydrogel-based construct was successfully fabricated using type II collagen crosslinked with the PEG system, enriched with HA via micro-dispensing methods, as shown in Figure 2A. The crosslinker, 4S-StarPEG molecule is a pegylated structure presenting four terminal N-hydroxysuccinimidyl (NHS) reactive groups. NHS-terminal groups react with the amine groups of type II collagen to initiate *in situ* crosslinking, thereby forming the hydrogel. The 3D spherical-shaped hydrogels with the macromolecule weight ratio of HA and COLII at 1:9 and 4.5:9 was maintained as physical structures after a complete crosslinking reaction (Figures 2B, C).

The aim of swelling analysis is to study the water intake capacity of the hydrogel in an aqueous solution. When compared between the dry and wet state of hydrogel, the swelling percentage increased to 341% and 469% in HA/COLII 1:9 hydrogel at 24 and 48 h; however, the swelling capacity decreased to 111% at 144 h later. HA/COLII 4.5:9 hydrogel showed a similar pattern of swelling behaviour, which drastically increased to 934% and 910% at 24 and 48 h, and it declined to 418% after 144 h. Thus, between both formulations, HA/COLII 4.5:9 hydrogel showed a significantly higher swelling capacity in comparison to HA/COLII 1:9 hydrogel, suggesting that a more significant amount of HA is capable of absorbing a large amount of water *in vitro* (Figure 2D).

**FIGURE 3:**
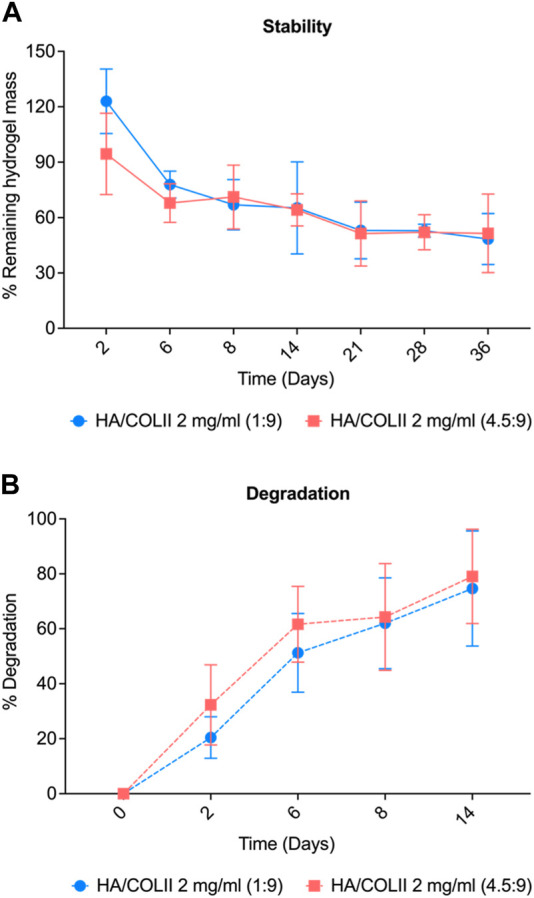
Physical characterisation of hydrogel for stability and degradability profile. **(A)** Stability of the hydrogel in PBS. **(B)** Degradation profile of HA/COLII hydrogel in type II collagenase over 14 days *p < 0.05, significant difference between groups, by two-way ANOVA and Bonferroni’s *post hoc* test. Data are means ± SEM, *n* = 3.

For the stability study, HA/COLII 1:9 hydrogel incubated in PBS showed a decrease in weight from 122% to 77% before it gradually decreased at 65% to reach the plateau. HA/COLII 4.5:9 hydrogel reduced 94% and 64% weight at day 2 until 14 and remained stable until day 36. Both hydrogels showed no significant difference in weight over time. This result indicates that both hydrogel formulations are stable to the hydrolytic process in an aqueous solution over a month (Figure 3A).

For *in vitro* degradation, HA/COLII 1:9 hydrogel incubated in collagenase showed a substantial increase of degradation from 20% to 51% on day 2 until day 6 and gradually increased to 74% at a later time-point, day 14. HA/COLII 4.5:9 hydrogel showed an increase in degradation from 32%, 61%, and 79% on day 2, day 6, and day 14, which was slightly higher degradation when compared to that HA/COLII 1:9 hydrogel, but no significant difference between groups. This finding suggests that HA/COLII 1:9 hydrogel is more resistant to enzymatic reactions *in vitro* (Figure 3B).

**FIGURE 4:**
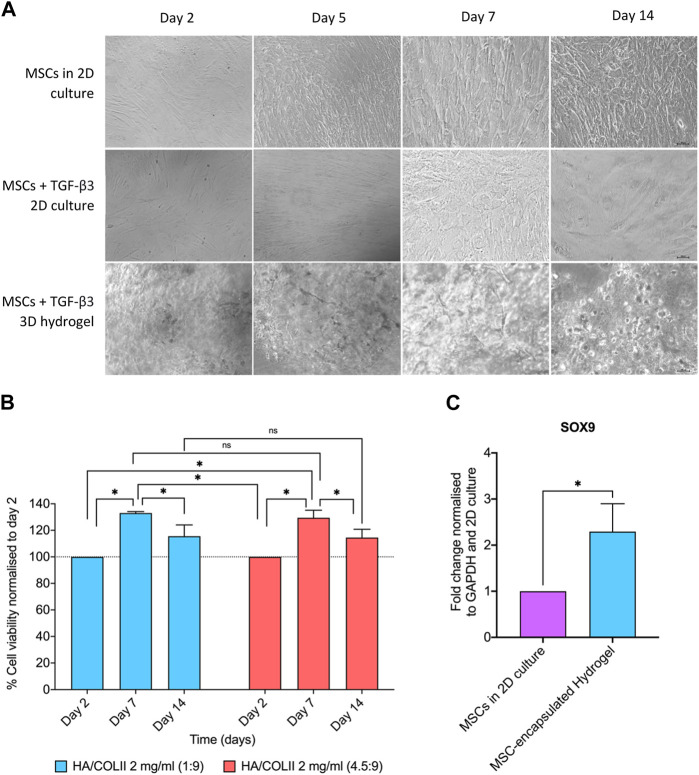
The cell morphology, viability and NP phenotypic markers of hWJ-MSCs encapsulated in HA/COLII hydrogel. **(A)** Light microscopy showed WJ-MSCs encapsulated in HA/COLII hydrogel until 14 days in culture. **(B)** Viability of hWJ-MSCs encapsulated in HA/COLII hydrogel over 14 days in culture. The percentage of cell viability was plotted for each group on days 2, 7, and 14. **(C)** Upregulation of SOX9 mRNA expression was observed on hWJ-MSCs encapsulated in HA/COLII hydrogel, indicating for NP phenotypic marker. *p < 0.05, the significant difference between groups, by two-way ANOVA for viability and *t*-test for gene fold change, and Bonferroni’s *post hoc* test. Data are means ± SEM, *n* = 3; scale bar, 100 μm.

We then incorporated hWJ-MSCs into the optimal hydrogel formulation with HA/COLII ratio of 1:9 and cultured them in Transforming Growth Factor-β3 (TGF-β3) supplemented media up to 14 days. Light microscopy showed fibroblastic-shaped hWJ-MSCs on 2D culture with and without TGF-β3 supplementation. When compared to the 3D culture of hydrogel, a homogenous cellular distribution was observed on hWJ-MSCs encapsulated in the 3D microenvironment of HA/COLII hydrogel up to 14 days in TGF-β3 supplemented culture. In addition, the cells were round morphology and individually distributed within the hydrogel system, indicating that hWJ-MSCs have been differentiated into NP-like cells, which resemble the morphology of NP cells (Figure 4A).

For the viability study, hWJ-MSCs encapsulated in HA/COLII hydrogel demonstrated an increased percentage of viability at days 7 and 14 when compared to day 2, with no significant difference between all hydrogels. This result suggests that the 3D microenvironment of HA/COLII hydrogel is non-cytotoxic and becomes favourable for cells to survive and proliferate *in vitro* (Figure 4B).

A higher mRNA expression of SRY-Box Transcription Factor 9 (SOX9) was observed in the hWJ-MSC-encapsulated hydrogel when compared to MSCs in 2D culture. This result suggests that cell differentiation of hWJ-MSCs into NP-like cells is confirmed by the expression of SOX9, an NP phenotype marker (Figure 4C).

## Discussions

In tissue engineering and regenerative medicine, encapsulating Wharton’s jelly-derived mesenchymal stem cells (WJ**-**MSCs) within a hydrogel matrix is a standard procedure. The body’s extracellular matrix (ECM) is mimicked in three dimensions (3D) by hydrogels, which promote cell survival, proliferation, and differentiation. To assure the quality, effectiveness, and compatibility of the fabricated 3D hydrogel, characterization is an essential stage in the production process. It entails assessing and comprehending the hydrogel’s swelling capacity, stability, degradation, and biological properties. Crosslinking method is vital as it provides stability and mechanical integrity to the hydrogel, allowing it to retain its shape and provide a suitable environment for encapsulated cells or bioactive agents (Hennink and van Nostrum, 2012). In this study, 4S-StarPEG was used to help in the cross-linking of the amine groups of the type II collagen. Due to its biocompatibility, adjustable characteristics, and capacity to construct hydrophilic networks, PEG is a versatile polymer frequently used to fabricate hydrogels (Shi et al., 2021). The 2 ratios of the successfully fabricated hydrogel with HA/COLII at 1:9 and 4.5:9 was further compared to its characteristics. Swelling study not only can help in understanding the capabilities or behaviour of the hydrogel, but it can also provide insight on its biocompatibility interactions towards cells. Based on the intended use and desired features, the ideal swelling percentage for a given hydrogel can be determined. It frequently entails striking a balance between maintaining optimal mechanical and functional qualities and maximising swelling capacity for adequate water absorption. Based on the result in Figure 2D, the difference in swelling capacity (%) was affected by the concentration of HA. The higher the concentration of HA, the higher the swelling capacity. When HA is included in a hydrogel matrix, it increases the system’s overall hydrophilicity, which is suitable for hydrated tissue such as NP in the IVD. Due to HA’s propensity to absorb and hold water, its presence can improve the hydrogel’s ability to absorb water and swell (Lai et al., 2016; Isa et al., 2023). The hydrogel may experience such severe swelling or de-swelling in reaction to changes in its surroundings that the phenomenon is known as volume collapse or phase changeover (Shin et al., 2010).

Next, the fabricated hydrogel for both formulations exhibits excellent stability undergoing hydrolytic degradation through immersion and incubation with PBS. The mass of the remaining hydrogel for both groups reached the plateau after 21 days indicating their stability to prevent further degradation and equilibrium swelling. At 37°C, the hydrogel is subjected to an environment that mimics the physiological conditions of the human body. The equilibrium swelling reached after 20 days indicates that the hydrogel has absorbed the maximum amount of water or PBS under these conditions. It implies that the hydrogels have achieved a stable state of swelling and maintain their mass over an extended period. The excellent stability of the hydrogel can also be concluded due to the crosslinking with 4S-StarPEG, which provides stability in sensitive temperatures and osteogenic functions (Wieland et al., 2007; Sun et al., 2023). This stability is significant for applications that require the hydrogel to maintain its physical integrity, swelling properties, and other characteristics over an extended duration. Hydrogels will experience fatigue under cyclic stress, which is the gradual loss of material characteristics or the initiation and propagation of cracks that may result in a material fracture (Bai et al., 2019). It suggests that the hydrogel is suitable for use in environments that approximate the conditions of the human body, as it remains structurally intact and retains its desired properties over a prolonged period. In correlation to the degradation study in Figure 3B, the stability of the hydrogels differs in the enzymatic degradation in which, with the presence of collagenase, the degradation percentage increases over time (14 days). However, the two groups have no significant differences, although the HA/COLII 1:9 showed a slightly lower degradation percentage.

Generally, the hWJ-MSCs have fibroblasts-like adherent cells that are wide, flat and polygonal in shape (Xie et al., 2015). MSCs in 2D cell culture showed the properties of the cells being elongated in shape following the flat surface. In hydrogel, the morphology of the cells can be affected by the stiffness of the hydrogel, where the cells may appear in spherical and rounded morphology in harder/stiffer hydrogel (Tong et al., 2016; Crosby et al., 2022). As shown in Figure 4A, the hWJ-MSCs are rounded and spherical and evenly distributed, at most at day 14, in the hydrogel, indicating the hWJ-MSCs had been differentiated into NP-like cells. The morphology could also be affected by the cell density and the hydrogel formulation (Mohand-Kaci et al., 2013). The cell viability was further evaluated and compared between the two groups. Both groups showed excellent cell viability (%), which is more than 100%. It is important to note that the cell viability study was done via MTT assay. The astounding cell viability percentage can be due to the cells actively dividing and proliferating, indicating that the 3D microenvironment of HA/COLII hydrogel mimics the ECM and becomes favourable for the cells. Dynamic culture conditions have been hypothesised to enhance oxygenation and molecule diffusion within the scaffolds, possibly enhancing cell viability (Zhang et al., 2009; El-Ayoubi et al., 2011). Other prominent factors of increased cell viability include the materials used in fabricating the hydrogel. HA and collagen provide a perfect mimic to ECM for the hWJ-MSCs. HA can accelerate and improve cell migration and proliferation rates (Della Sala et al., 2022) while collagen has demonstrated exemplary performance in forming native-like NP ECM *in vitro*, to mimic the ECM (van Uden et al., 2017).

The SOX9 mRNA was expressed higher in the hydrogel compared to the 2D cell culture. SOX9 is one of the NP phenotypic markers. Increased expression of SOX9 mRNA indicates the cell differentiation of hWJ-MSCs into NP-like cells following activating the TGF-β3 pathway. The hydrogel is composed of HA and COLII, creating an ECM-based microenvironment to mimic the predominant ECM content of native NP tissue. Thus, hydrogel provides a conducive microenvironment for MSC differentiation towards NP-like cells. A previous study reported that human pluripotent stem cells have been differentiated into notochord-like and NP-like cells after activating the TGF-β3 pathway for up to 2 weeks in culture (Zhang et al., 2020). The hydrogel’s spatial organisation and cell-cell interactions promote localised microenvironments or signalling gradients, which explain the upregulation of SOX9 mRNA (Khetan and Burdick, 2011). Additionally, the hydrogel’s supporting matrix containing HA and collagen promotes the production and deposition of extracellular matrix (ECM), which raises the expression of SOX9 (Jiang et al., 2023). TGF-β3 has also been reported to positively impact the protein SOX9’s stabilisation ability, supporting this study (Chavez et al., 2017). Alternatively, a microenvironment that promotes the upregulation of SOX9 mRNA expression may be created by the mechanical qualities of the hydrogel, such as stiffness or elasticity, which can affect gene expression (Ledo et al., 2020).

Collectively, we have shown the development of HA/COLII-based hydrogel by characterising the biomacromolecule concentration results in an optimal swelling capacity, stability, degradability, and non-cytotoxicity in nature. This hydrogel effectively mimicked the NP microenvironment, guiding human hWJ-MSCs toward NP-like cells.

These findings may imply the use of hydrogel tailors to the severity of IVD degeneration to enhance the efficacy of the treatment. This hydrogel can potentially be used as a therapeutic biomaterial and advanced cell delivery system for IVD tissue engineering towards a precision medicine approach (Mohd Isa et al., 2022). For early or mild IVD degeneration, hydrogel could be utilised by creating IVD-specific matrices by mimicking native ECM of IVD to maintain cellular phenotype as well as designing hydrogel with therapeutic biomaterial such HA as a potential therapeutic candidate in targeting mechanisms underlying IVD degeneration in the treatment of low back pain. In the clinical setting, this hydrogel could be utilised as a minimally invasive procedure through percutaneous intradiscal injection, which eliminates the need for repetitive surgeries. In addition, the HA molecule in hydrogel composition has been reported to exhibit anti-inflammatory and anti-nociceptive effects in IVD degeneration, which targets the inflammation and pain mechanism underlying IVD degeneration (Mohd Isa et al., 2018b) aiming to alleviate pain and avoid surgery complications such as degeneration of adjacent IVDs, all of which could improve patient quality of life postoperatively. Furthermore, the hydrogel could be employed as an advanced cell delivery system by incorporating WJ-MSCs into a hydrogel system that could be tailored to an advanced stage of IVD degeneration to restore the IVD’s cellularity and facilitate tissue regeneration where the implanted or injected cells into the IVD could synthesise the new ECM for tissue regeneration, which in turn impacts the cellular function. These cells can be administered individually or via injection or implantation within a biodegradable hydrogel.

## Conclusion

Fine-tuning HA/COLII-based hydrogel by varying the biomacromolecule concentration provides the optimal swelling capacity, stability, degradability, and non-cytotoxic, thus mimics the NP niche in guiding hWJ-MSCs towards NP-like cells, and potentially can be used as an advanced cell delivery system for intervertebral disc regeneration.

## Methods

### Fabrication of 3D HA/COLII hydrogel

The hydrogel-based 3D construct was fabricated using bovine articular-derived type II collagen (COLII) solution (Reprocell, United States) at 2 mg/mL enriched with a high molecular weight of sodium hyaluronate (HA) (Lifecore Biomedical, United States) 10 mg/mL at various weight ratio of HA to COLII was 1:9 and 4.5:9. To chemically crosslink the hydrogel, polyethylene glycol (4S-StarPEG) (JenKem Technology United States Inc.) molecular weight 10,000 was added in mixture solution with a 1:1 M ratio. Then, it was micro-dispensed as 10 μL droplets on a hydrophobic surface of a modified glass slide to obtain a 3D spherical-shaped hydrogel before incubation at 37°C for 1 h to complete the crosslinking reaction.

### Assessment of swelling capacity

To determine swelling behaviour, pre-dry weighted (W_d_) hydrogels were incubated in 1 mL of phosphate buffer saline (PBS) at 37°C before measuring the hydrogel’s wet weight (W_1_) at 24, 48, and 166 h. The percentage (%) of swelling was calculated using the formula:

$$\frac{W_{1}–W_{d}x100}{W_{d}}$$

### Assessment of degradation and stability

To observe degradation, the hydrogels were incubated in 1 mg/mL of type II collagenase (Worthington, United States) at 37°C for 2, 6, 8, and 14 days. In contrast, stability was observed by incubating hydrogels fully submerged in PBS at 37°C for 2, 4, 8, 14, 21, and 36 days. The degradation medium was replenished with a freshly prepared enzyme solution every 2 days. The initial wet weight (W_0_) and wet weight (W_1_) of hydrogels were measured. The percentage of stability was calculated using the remaining hydrogel mass using the formula:

$$\frac{W_{1}x100}{W_{0}}$$

The percentage of degradation was calculated using the formula:

$$\frac{W_{1}–W_{0}x100}{W_{0}}$$

### Microencapsulation of hWJ-MSCs in HA/COLII hydrogel

The hWJ-MSCs were seeded at a density of 1.5 × 10^5^ cells/cm^2^ in a tissue culture flask T75 before incubation at 37°C, 5% CO_2,_ and 90% humidity. Media was changed every 3 days until cell confluent around 70% before use. The cells were encapsulated in the 3D HA/COLII hydrogel at density 0.8 × 10^6^. To encapsulate the cells in the hydrogel, the cell suspension was introduced after adding the crosslinker to the mixture solution during the procedure. Then, the cell encapsulated in the hydrogel was incubated for an hour at 37°C to allow complete crosslinking. The cell-encapsulated hydrogel was transferred to the tissue culture plate containing complete media supplemented with 10 ng/mL of TGF-β3 (Elabscience Biotechnology Co., Ltd., China). The construct was cultured for up to 14 days to allow differentiation of hWJ-MSCs towards NP-like cells. The media was changed every 3 days.

### Assessment of cell morphology and viability

After 14 days in culture, the morphology of hWJ-MSCs encapsulated in the hydrogel was observed under the light microscope. The viability of cells encapsulated in the hydrogel was determined using the 3-(4,5-dimethylthiazol-2-yl)-2,5-diphenyltetrazolium bromide (MTT) assay (Merck, Germany). The percentage of viability was calculated based on the absorbance (Optical Density (OD)) of each experimental group. The percentage of viability was calculated and normalized to day 2 control using the formula:

$$\frac{\text{Mean}\;\text{ODx}100}{\text{Mean}\;\text{OD}\;\text{control}\;\text{day}\;2}$$

### NP phenotypic marker by RT-qPCR

After a fourteen-day MSC-encapsulated hydrogel in culture, samples were homogenized in 1 mL of TRIzol reagent using TissueLyser (Qiagen). Total RNA was extracted from MSCs using TRIzol reagent (Invitrogen) and NucleoSpin RNA kit (Macherey-Nagel) following the manufacturer’s protocol. RNA was reverse transcribed by RevertAid™ First Strand cDNA Synthesis Kit (Thermo Scientific) in a 20 μL reaction mixture using CFX96 Touch Real-Time PCR Detection System (Biorad, United States). The cDNA products were amplified using Maxima SYBR Green qPCR Master Mix (Thermo Scientific) and following SOX9 primer (Hs.PT.58.38984663). PCR reactions were conducted in triplicate using CFX96 Touch Real-Time PCR Detection System (Biorad, United States of America). The results were analyzed using the 2^−ΔΔCT^ method and presented as fold change (relative gene expression) normalized to the GAPDH gene (Hs.PT.39a.22214836).

## Statistical analysis

Statistical differences were analyzed by GraphPad Software, using one-way ANOVA for analyses of the viability, swelling, stability, degradation, and *t*-test for PCR. All ANOVAs were further evaluated with Bonferroni *post hoc* analysis, and *p < 0.05 was deemed statistically significant. All error bars indicate the standard error of the mean (S.E.M).

## Data Availability

The raw data supporting the conclusions of this article will be made available by the authors, without undue reservation.
